# A Framework for the Testing and Validation of Simulated Environments in Experimentation and Training

**DOI:** 10.3389/fpsyg.2020.00605

**Published:** 2020-03-31

**Authors:** David J. Harris, Jonathan M. Bird, Philip A. Smart, Mark R. Wilson, Samuel J. Vine

**Affiliations:** ^1^School of Sport and Health Sciences, University of Exeter, Exeter, United Kingdom; ^2^Centre for Simulation, Analytics and Modelling, University of Exeter Business School, Exeter, United Kingdom

**Keywords:** fidelity, presence, training, transfer, validity, virtual reality

## Abstract

New computer technologies, like virtual reality (VR), have created opportunities to study human behavior and train skills in novel ways. VR holds significant promise for maximizing the efficiency and effectiveness of skill learning in a variety of settings (e.g., sport, medicine, safety-critical industries) through immersive learning and augmentation of existing training methods. In many cases the adoption of VR for training has, however, preceded rigorous testing and validation of the simulation tool. In order for VR to be implemented successfully for both training and psychological experimentation it is necessary to first establish whether the simulation captures fundamental features of the real task and environment, and elicits realistic behaviors. Unfortunately evaluation of VR environments too often confuses presentation and function, and relies on superficial visual features that are not the key determinants of successful training outcomes. Therefore evidence-based methods of establishing the fidelity and validity of VR environments are required. To this end, we outline a taxonomy of the subtypes of fidelity and validity, and propose a variety of practical methods for testing and validating VR training simulations. Ultimately, a successful VR environment is one that enables transfer of learning to the real-world. We propose that key elements of psychological, affective and ergonomic fidelity, are the real determinants of successful transfer. By adopting an evidence-based approach to VR simulation design and testing it is possible to develop valid environments that allow the potential of VR training to be maximized.

## Introduction

How real is virtual reality? This question raises weighty metaphysical issues, but it also poses a very practical challenge for scientists seeking to use virtual reality (VR) technologies for experimentation and training. A simulation aims to reproduce some aspects of a task (e.g., perceptual information and behavioral constraints) without reproducing others (e.g., danger and cost; Gray, 2002; Stoffregen et al., 2003). Consequently, understanding the degree of concordance between the simulated environment and the corresponding real-world task is essential for the successful application of VR, in both the psychology lab and in the field. While this is a challenging endeavor on its own, there is also considerable confusion within cognitive science about terms like *fidelity, validity, immersion* and *presence* and how VR environments should be evaluated. For example, environments are often judged to be “high fidelity” if they provide a detailed, realistic visual scene, but the superficial appearance may have little relationship with functionality, especially in the context of VR for education and training (Carruth, 2017). In effect, the distinction between presentation and function is often overlooked.

For VR to be implemented more effectively as a training tool, greater conceptual clarity is imperative, and more rigorous ways of testing and validating simulations must be developed. In this review, we aim to address some of this confusion by, firstly, addressing some conceptual issues and outlining a taxonomy of fidelity and validity, and secondly, by proposing evidence-based methods for establishing fidelity and validity during simulation design. Here we particularly focus on VR for training perceptual-motor skills, such as for applications to sport, surgery, rehabilitation and the military – the kind of active skills which may be particularly suited to VR training (Jensen and Konradsen, 2018). However, these principles may also apply to many uses of VR as a training tool, and as such, we hope to provide a framework for those seeking to develop more effective, evidence-based VR simulations.

## Immersion and Presence

Immersive VR is an alternate world composed of computer-generated sounds and images with which the user can interact using their sensorimotor abilities (Burdea and Coiffet, 2003; Slater and Sanchez-Vives, 2016). The proliferation of technologies for both using and creating augmented reality (AR), mixed reality (MR), and VR experiences has led to rapid adoption of VR as a training tool within human factors (Gavish et al., 2015), sport (Bird, 2019), rehabilitation (Levac et al., 2019), and surgery (Hashimoto et al., 2018). The lure of new technologies for training is sufficiently great that these methods have been applied before there is a foundational understanding of how to optimally implement VR training (albeit with some successes, e.g., Calatayud et al., 2010; Gray, 2017). Particular issues yet to be addressed include: the determinants of effective transfer of training (Rose et al., 2000; Rosalie and Müller, 2012); the requisite levels of fidelity and validity and how to test them (Gray, 2019); and the effect of VR on basic cognitive and perceptual processes (Harris et al., 2019d).

As a means of reducing the aforementioned confusion surrounding key terminology, we adopt Slater and Sanchez-Vives (2016) definition of *immersion* as the technical capability of a system that allows a user to perceive the virtual environment through natural sensorimotor contingencies. While immersion is an objective feature of the input provided to the user, the subjective experience that is created of actually being inside the virtual environment is termed *presence* (Baños et al., 2004; Bowman and McMahan, 2007). Slater (2009) and Slater and Sanchez-Vives (2016) suggest that there are two important components to the experience of presence; *place illusion* (the illusion of “being there” in the virtual environment), and *plausibility* (that the depicted scenario is really occurring). A consequence of place illusion and plausibility is that users behave in VR as they would do in similar circumstances in reality (Slater and Sanchez-Vives, 2016), which is of paramount importance for VR training. Despite users knowing that the virtual environment is fictitious (Stoffregen et al., 2003), researchers have suggested that presence can prompt users to feel anxious near illusory drops (Meehan et al., 2002), maintain social norms with virtual others (Sanz et al., 2015), and exhibit stress when forced to cause harm to avatars (Slater et al., 2006).

For training and experimentation purposes the virtual environment needs to be only as “real” as is required for achieving the desired learning outcome, be that training perceptual-motor skills (Tirp et al., 2015), habituating to stress inducing stimuli (Botella et al., 2015) or studying sensorimotor processes (Buckingham, 2019). However, differing target populations may need to be engaged in different ways to produce similar learning outcomes. For VR to be effective in a training context, there must be a correspondence between key elements of the real and virtual tasks that are functional for task learning. Other elements such as graphical realism are often inconsequential in comparison (Dahlstrom et al., 2009). For instance, when using VR to study the perceptual information that informs catching a ball, there is no requirement that the ball looks realistic, the scene is highly detailed, or the task is particularly immersive (see e.g., Fink et al., 2009; Zaal and Bootsma, 2011). Nonetheless, realistic kinematic and depth information pertaining to the ball are necessities. It is elements of the simulation such as these that determine the *fidelity* and *validity* of virtual environments. In the remainder of the article we outline various types of fidelity and validity, how they can be assessed, and how they contribute to effective transfer of training (see Table 1).

**TABLE 1:** Summary of validity and fidelity terminology.

**Term**	**Description**	**How to test**
*Face validity*	Does the simulation look and feel realistic?	Self-reports from users concerning plausibility
*Construct validity*	Does the simulation provide an accurate representation of real task performance?	Ability of the simulation to distinguish real-world experts from novices and track improvements
*Physical fidelity*	Is there a high degree of detail and realism in the physical elements of the simulation?	Participant reports of realism and measures of presence (both self-report and psychophysiology)
*Psychological fidelity*	Does the simulation accurately represent the perceptual and cognitive features of the real task?	Measurement and comparison of mental effort, gaze behavior, neural activity etc., between real and virtual tasks
*Affective/emotional fidelity*	Does the simulation elicit emotional responses (e.g., stress or fear) in a similar way to the real task?	Self-reported experiences of users or online monitoring of psychophysiological indices of affect
*Ergonomic/biomechanical fidelity*	Does the simulation elicit realistic motor movements?	Assessing the realism of VR movement parameters through motion tracking, and comparing amplitude, speed, inter-joint coordination etc., with real actions

## Transfer of Training

The capacity to effectively apply and adapt learning in the face of constant environmental variation (i.e., transfer) is fundamental to many human activities (Rosalie and Müller, 2012). Transfer of training occurs when prior experiences in a particular context can be adapted to similar or dissimilar contexts (Barnett and Ceci, 2002). Ultimately, the test of a successful VR training simulation is the degree to which skills learned in the virtual environment can be applied to the real-world. Classical theories of learning, like Thorndike’s (1906) identical elements theory (later developed into Singley and Anderson’s (1989) identical productions model) support the notion that successful transfer is contingent on the coincidence of stimulus or response elements in learning and transfer contexts^1^, suggesting that only near transfer may be possible. For instance, accurate size estimation of geometric shapes is dependent on specific learning with objects of similar size and shape (Woodworth and Thorndike, 1901).

The foremost competing paradigm to similarity-based transfer is principle-based transfer theory (Judd, 1908), which focuses on the coherence of principles, rules, or laws between settings, irrespective of superficial contextual variation. This approach proposes that learning can more easily be generalized, provided equivalent principles or rules are present in learning and transfer contexts. Achieving far transfer of learning that is generalized across domains which are only loosely related to each other is, however, notoriously difficult to achieve (Sala and Gobet, 2017). Nonetheless, VR training generally does not aim to achieve domain general improvements. Instead, as VR aims to recreate the performance environment, near transfer between tightly coupled domains is the goal, such as is common across human learning. The challenge facing the field of VR training is to establish whether a simulation is realistic enough, and how to enhance the aspects of realism that really matter for effective transfer. We believe that a better understanding of fidelity and validity in the design and testing of VR environments can help to meet this challenge.

## Validity

In a general sense, validity is the extent to which a test, model, measurement, simulation, or other reproduction provides an accurate representation of its real equivalent. For example, a valid measurement truly represents the underlying phenomenon it claims to measure. Similarly, a valid simulation is one that is an accurate representation of the target task, within the context of the learning objectives and the target population. This is not to say that the simulation is *the same*. A simulation aims to capture key features of the real task and environment, rather than exactly emulate or imitate it. A number of types of validity are discussed in relation to measurement methods in Psychology (e.g., criterion and concurrent validity) but there are two primary types to consider in simulation design; *face validity* and *construct validity* (see Table 1).

### Face Validity

Face validity is the subjective view users have of how realistic a simulation is. Accordingly, face validity may be an important contributor toward perceptions of plausibility (Slater, 2009). Face validity is often highly dependent on the superficial visual features of the simulation (see section “Physical Fidelity” below) but is also influenced by structural and functional aspects, such as how user input relates to actions. Consequently, the design of the simulation and the technical capabilities of the system (i.e., immersion) are important determinants of face validity. Within a learning context, face validity is important in one sense, and irrelevant in another. It can be important because it correlates with up-take and is often needed to achieve buy-in, which can derail training if not achieved. Conversely, it is also irrelevant because it likely has no correlation with actual learning. A simulation can have face validity and be useless; a simulation can have no face validity at all and be an excellent training tool (Dahlstrom et al., 2009). As discussed, theories of transfer propose that a coincidence of stimulus and response elements, or underlying principles between the practice and target tasks is required for transfer of learning. Face validity is unlikely to be a good indicator of whether any of these conditions are met.

Assessment of face validity often relies on participant reports and verbal feedback about whether the simulation is a good representation of the real task, either formally or informally (e.g., Bright et al., 2012). Collecting participant feedback regarding face validity is a commonly used approach in the field of surgical simulation. In this context, the opinions of expert surgeons are often sought about how the simulation looks and feels and is an important part of simulation validation (Sankaranarayanan et al., 2016; Roberts et al., 2017). A similar approach is also used in the development and testing of aircraft simulations with expert pilots (Perfect et al., 2014). In many other contexts face validity is not explicitly tested, but remains an implicit factor in simulation adoption. Consequently, face validity may well be a hurdle to overcome, but not a major contributor to training success.

### Construct Validity

Construct validity exists in a more objective sense than face validity, and is the extent to which the simulation provides an accurate representation of the real task. As a result, it is crucial for achieving transfer of learning. Many simulations are used to track learning, or to index proficiency on a task, which depends on some level of functional similarity between the simulation and the real task. A simulation with good construct validity should be sensitive to variation in performance between individuals (e.g., real-world novices and experts) and within individuals (e.g., learners developing over time), as this would indicate a coherence of principles, rules or stimulus and response elements between the real and VR task. These fundamental similarities are likely required for transfer of training.

*Predictive validity* refers to the ability to reliably predict future performance outcomes and is closely related to construct validity. If the simulated task replicates some core aspect of the real skill (i.e., good construct validity), then simulation performance is more likely to accurately predict future real-world performance^2^. One possible application of VR simulations is to serve as a tool for recruitment or selection (e.g., Moglia et al., 2014). Evaluating the aptitude of trainees in VR is particularly appealing when assessment on the real task would be impractical or dangerous, such as in military or surgical settings. For such purposes, predictive validity must be established to ensure that selection decisions based on simulation performance are reliable.

There are clear opportunities for testing construct validity through expert versus novice comparisons and sensitivity to practice-induced improvements. This validation method has previously been adopted by Harris et al. (2019a) when comparing putting performance of novice and elite-level golfers in a VR golf simulation, and by Bright et al. (2012) when comparing tissue resected when using a minimally invasive surgical simulation. Not only did the real-world experts resect more tissue than the novices in the simulation, but the simulation was sensitive to practice induced improvements in the novices (Bright et al., 2012, 2014). Measuring inter- and intra-individual variation is an effective method for demonstrating construct validity, but to achieve it in the first place requires an understanding of the competencies and skills of the to-be-trained task, and ensuring that the rules, interactions and criteria of the real-task have one-to-one counterparts in the simulation.

## Fidelity

Fidelity is the extent to which a simulation recreates the real-world system, in terms of its appearance but also the affective states, cognitions or behaviors it elicits from its users (Perfect et al., 2014; Gray, 2019). To achieve construct validity (and effective transfer) it is necessary to ensure there is a sufficient level of fidelity in relevant aspects of the simulation. For instance, when implementing VR as a training tool for perceptual-motor skills it may be important to assess fidelity for eliciting affective states like stress, for directing attention to relevant information, and allowing for movements representative of the real skill. However, the fidelity of the simulation must be assessed in relation to training goals. Gray (2002) propose that a simulation can only be considered “high-fidelity” in relation to the research question being asked, and the same holds true for training. A simulation that is developed to train a motor skill (e.g., a golf swing) would be required to elicit realistic actions, but realistic ergonomics might be irrelevant for a simulation that is developed to enhance proficiency of a purely cognitive task.

### Physical Fidelity

Physical fidelity refers to the level of realism provided by the physical elements of the simulation; primarily visual information (including field of view) as the principal sensory modality in VR, but also realistic behavior of objects, adherence to the normal laws of physics, and level of functionality. As is the case for face validity, the physical fidelity of the environment is likely to be important for eliciting a feeling of presence in the participant, and in particular creating the illusion of plausibility (Slater, 2009), which will depend heavily on the immersion of the technology. If basic elements of physical fidelity are low, such as allowing the user to walk through walls, the illusion will be broken. When the term “high fidelity” is used in relation to simulations, it is generally in reference to high physical detail, but as we outline below, realistic sights and sounds are just one element of a high-fidelity simulation.

While high graphical realism is likely to increase motivation to engage with simulation training and adds to the “wow factor” of VR (a highly positive, but superficial response), it is unlikely to be what creates effective transfer. For instance, in the context of sport, high graphical realism would seem to be important, but sporting performance is dependent on the efficient use of only a subset of the available perceptual information (Davids et al., 2005), making much of the detail irrelevant for training. One instance where physical realism may be important is in using VR to acclimatize performers to a particular environment or for a VR equivalent of mental imagery or visualization (e.g., Sorrentino et al., 2005). Other than adherence to normal physical laws, high fidelity physical features will often not be the stimulus-response correspondences or underlying principles that determine transfer, as outlined in theories of transfer. Nonetheless, very low physical fidelity may still be a barrier to effective training if it lowers motivation to use the simulation.

One method for assessing physical fidelity is through either direct participant reports or measurements of presence (e.g., Harris et al., 2019a). As presence is a result of achieving a sufficient level of fidelity, this is an indirect measure, but high levels of presence would indicate that the physical realism is sufficient to make the simulation believable (i.e., plausibility) and induce the feeling of “being there” (i.e., place illusion). Presence seems to be important for increased engagement in virtual training (Stanney et al., 2003), and can be measured either through self-report (Usoh et al., 2000) or online using psychophysiological indices like eye-movements, electroencephalography (EEG) and heart rate (Jennett et al., 2008; Nacke and Lindley, 2008). Whether higher levels of presence are beneficial for learning beyond the effect of enhanced engagement remains to be established (Dahlstrom et al., 2009; Fowler, 2015; Gray, 2019), and is an important question for future research.

### Psychological Fidelity

Psychological fidelity is the degree to which a simulation replicates the perceptual-cognitive demands of the real task (Gray, 2019). For instance, a high-fidelity driving simulation should require the participant to attend to similar areas of the scene (e.g., other cars, pedestrians, street signs) and demand a similar level of attentiveness and effort as if they were engaged in real-world driving. Accordingly, practice in this simulation should also lead to the development of psychological skills germane to real driving, such as learning to attend to the most informative areas of the road and predict the behavior of other traffic. Developing psychological skills like these would likely support real-world transfer.

Particular considerations for psychological fidelity include determining whether individuals: exhibit similar gaze behavior in real and virtual tasks (e.g., Vine et al., 2014); use similar perceptual information to control their actions (e.g., Bideau et al., 2010); and experience similar levels of cognitive demand (e.g., Harris et al., 2019a). Achieving place illusion will support these aspects of fidelity. For applications to domains like sport, surgery, and the military that place demands on perceptual-cognitive skills, psychological fidelity may be one of the most important prerequisites for developing an effective simulation. Encouragingly, perceptual-cognitive skills have been shown to be transferable between closely related sports (Abernethy et al., 2005; Causer and Ford, 2014), supporting the notion that VR environments with good psychological fidelity should elicit positive transfer. While a number of studies have shown overall performance benefits as a result of VR training, few have directly addressed the development of perceptual-cognitive skills, such as the control of attention or anticipation (Tirp et al., 2015).

There are a number of ways to test psychological fidelity, including comparisons of mental effort, gaze behavior or neural activity between real and VR contexts. For instance, comparisons of gaze behavior between real and simulated surgery have previously been used to validate surgical simulations. For example, Vine et al. (2014) found that, in comparison to the VR task, during the real operation expert surgeons made more frequent, shorter duration fixations indicative of a less efficient visual control strategy. The authors suggested that the additional auditory and visual distractions, as well as the stress of the real operation, were responsible for the differences. Findings such as these highlight how many factors contribute to psychological fidelity in VR.

Evaluations of psychological fidelity can also address the mental and physical demands of the virtual task and compare them to the real task. For instance, Harris et al. (2019c) compared cognitive demands between real and virtual versions of a block stacking game (“Jenga”) using a self-report measure of task load, the SIM-TLX. Similarly, Frederiksen et al. (2020) compared cognitive load between head mounted immersive VR and standard screen presentation on a surgical training simulation, finding cognitive load (indexed by secondary task reaction time) to be significantly elevated in the immersive VR condition. Cognitive load is particularly relevant in the context of education and training as an optimal level of load is important for successful learning outcomes (Kirschner, 2002). If VR imposes additional load it could pose a challenge for training. Cognitive load could also be related to elements of user experience, like presence, although this was not the case in the aforementioned study of Harris et al. (2019c). In summary, assessments of psychological fidelity may require a combination of approaches, as well as an understanding of the perceptual-cognitive skills that are responsible for expert performance in the given task.

### Affective Fidelity

There has been considerable interest in VR applications for training tasks that are too dangerous to rehearse in the real-world (e.g., critical incidents in heavy industry) or for acclimatizing trainees to the high levels of stress they are likely to face in the field (e.g., defense and security). For these purposes, as well as for applications like treating anxiety disorders, a high level of affective fidelity is required (Moghimi et al., 2016). Affective or emotional fidelity requires the simulation to elicit a realistic emotional response in the user, such as fear, stress or excitement. The success of Virtual Reality Exposure Therapy (VRET), where exposure-based treatments for anxiety disorders are implemented in VR, indicate that realistic emotional responses can be achieved (Krijn et al., 2004; Morina et al., 2015). Similarly, Chirico and Gaggioli (2019) found that VR scenes elicited a range of emotions in a similar manner to the real thing. This realistic affective reaction relies on achieving the illusion of plausibility discussed by Slater (2009) and a sense of presence (Diemer et al., 2015), or no emotional response will occur. Familiarization with the emotion of anxiety can improve subsequent performance when anxious (Saunders et al., 1996). Hence, VR environments capable of eliciting some degree of emotional response may have significant benefits for preparing performers for pressurized environments (Pallavicini et al., 2016).

Affective fidelity can be easily assessed through self-report or psychophysiological measurement during the VR experience and compared to the real task. The clearest implementation of assessing affect in VR is in measuring stress during threatening VR experiences like Slater’s Milgram obedience (Slater et al., 2006) and the illusory pit room experiments (Meehan et al., 2002). Online measurements of cardiovascular activity (Ćosić et al., 2010), skin conductance (Meehan et al., 2002) and EEG alpha power (Brouwer et al., 2011) have all been employed as objective, online measures of stress induced by VR experiences. While strong stress responses have been elicited for phobic stimuli in VR, creating stress responses similar to those that will be experienced during high level sport, during complex surgical procedures or by defense and security personnel may be more challenging.

### Ergonomic and Biomechanical Fidelity

Ergonomic and biomechanical fidelity is the degree to which the VR environment allows for and promotes realistic movement patterns in the user, making the immersion of the system a major determinant of ergonomic fidelity. Despite advances in VR technology (e.g., haptic gloves, exoskeleton suits, muscle stimulation), the provision of realistic haptic information in VR remains a major challenge (Lopes et al., 2017). Haptic information is important for developing motor control, but if it is unavailable in VR, movements learned or performed in a simulation may differ from those learned in the real-world. Harris et al. (2019d) describe how the combined effect of artificial creation of depth (e.g., vergence-accommodation conflict; Kramida, 2016) and lack of end-point haptic information (Whitwell et al., 2015; Wijeyaratnam et al., 2019) may combine to force users toward a more deliberate mode of action control that is unlike real perceptual-motor skills. Further work is required to explore these potential limitations to learning and performing actions in VR, but the difference between real tennis shots and those performed in Nintendo Wii tennis provide a stark illustration of how widely action patterns can diverge. Biomechanical fidelity is a particular issue for applications to rehabilitation and sport, where low levels of fidelity could be actively disruptive if suboptimal motor patterns are reinforced in VR.

Assessment of biomechanical fidelity relies on motion tracking, and comparisons of movement amplitude, speed and inter-joint coordination between real and virtual environments. A number of attempts have been made to test the biomechanical and ergonomic features of VR environments, which have generally highlighted the difficulty in achieving this type of fidelity. Bufton et al. (2014) found that compared to real table tennis, participants in three different virtual table tennis games produced larger and faster movements when hitting the virtual ball. Similarly, in a simple reaching and grasping task Magdalon et al. (2011) found reaches were slower with wider grip apertures, and Covaci et al. (2015) found basketball shots made in a virtual environment had lower ball speed, higher height of ball release, and higher basket entry angle, compared with real basketball.

An important action to consider is walking, as VR is being widely adopted for studying and retraining gait (e.g., Young et al., 2011), but requires a VR-linked treadmill to allow users to move any appreciable distance. Work has already demonstrated biomechanical differences between VR and real walking (Mohler et al., 2007; Janeh et al., 2017), but the divergences from conventional movements could be related to the stationary locomotion methods (i.e., treadmills) rather than fundamental issues with traversing in the virtual environment. These differences seem to be a current limitation rather than prohibitive of gait retraining applications, and may well improve with technological advances (i.e., greater immersion).

## Practical Issues

**FIGURE 1:**
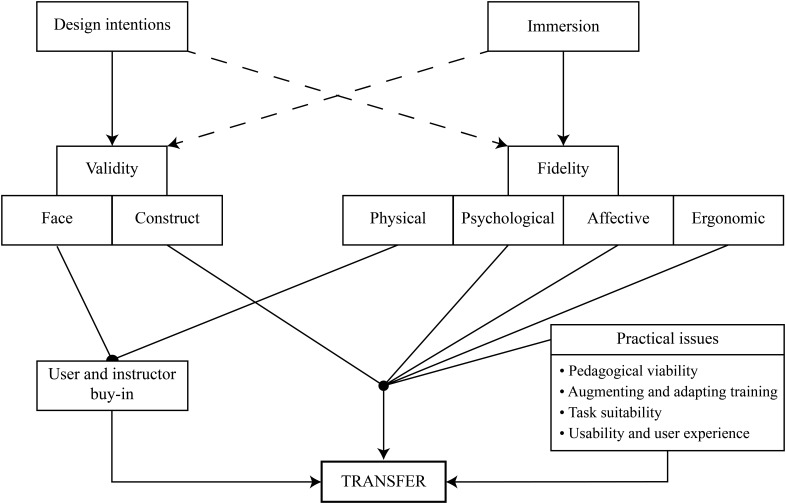
Taxonomy of fidelity and validity and successful transfer of learning from VR. We propose that construct validity and psychological, affective and ergonomic fidelity will have direct effects on successful transfer, while physical fidelity and face validity have indirect effects via the mediator user buy-in. Meanwhile practical and pedagogical factors will have both a direct and moderating effect on training outcomes (Blume et al., 2010). The degree to which validity and fidelity are achieved are a result of simulation design intentions and the capabilities of the technology. The degree of immersion of the technology is a key determinant of whether or not high levels of fidelity can be achieved. The design intentions also influence the level of fidelity and are particularly important for whether the simulation accurately represents the key elements of the real task in relation to training goals and audience (i.e., dashed lines indicate weaker proposed relationships).

In addition to establishing that a simulation is a valid recreation of the target task and is of sufficient fidelity to enable transfer of training, there are a number of practical concerns for effective implementation of VR training which may moderate the effectiveness of the simulation (see Figure 1), some of which we outline below (Carruth, 2017).

### Pedagogical Viability

An important pedagogical consideration is how design intentions correspond with training requirements. The importance of clear learning outcomes for designing and evaluating training has been well documented in the education literature (Kraiger et al., 1993). Successful training outcomes depend upon being able to articulate those aims for learners and design and create a simulation that supports them. A subsequent pedagogical issue is whether the simulation can be effectively adopted within a curriculum or training program to provide real learning benefits. Pedagogical viability is most often a pragmatic issue (e.g., *Where is the simulation? How is it used? How much does it cost?*), but can also be a conceptual and theoretical issue, according to your view of learning. The simulation is only a tool, and like any other must be appropriately periodized within the wider curriculum to achieve benefits. Important elements of instructional design should be considered to maximize the impact of simulation based training, such as levels of complexity and specificity in learning objectives, scaffolding of learning and evaluation of training (Kirschner, 2002; Carruth, 2017; Jensen and Konradsen, 2018).

### Augmenting and Adapting Training

One of the most compelling reasons to use VR for training is the possibility to augment and improve on existing practices with new methods, rather than just replacing them. Gray (2017) illustrates this well in the case of a baseball batting simulation. Virtual batting practice was found to outperform real batting practice, but only when the virtual version provided task demands constantly matched to the skill of the user. The complete control of the training space afforded by VR allows environmental constraints to be manipulated to improve skill acquisition (Renshaw et al., 2009). Guiding information, such as cues to important information or eye movement patterns of experts, can also be added to speed learning (Janelle et al., 2003; Vine et al., 2013). Other approaches include adaptive VR, which modifies the simulation to suit either the current performance level or psychophysiological state of the user (Moghim et al., 2015; Vaughan et al., 2016). An effective implementation of VR training makes full use of these possibilities.

### Task Suitability

The attraction of new technologies for training makes it easy to fall into the trap of overusing them. VR may allow more personalized and more accessible training in many instances, but is unlikely to be the best option when real-world practice is available. Even in the light of rapidly advancing VR technologies, the specificity of training principle highlights that to improve at, say catching a ball, there is one thing above all others that is likely to provide the greatest benefit – just catching a ball. For fine sensorimotor skills in particular, the unusual perceptual information in VR (lack of haptics and conflicting cues to depth) means that VR is not “real” enough to compete with real practice (Harris et al., 2019b). Hence, VR can be useful when the skill could otherwise not be practiced in the real-world (for practical or safety reasons), or when training can be *improved* in VR, but the rationale for using VR over other methods should be clear.

### Usability and User Experience

Much like face validity, user experience and usability is not a primary factor in training effectiveness, but may be a barrier to implementation and uptake. An otherwise high-fidelity simulation can be derailed if the user experience is poor. Issues like interaction and navigation in VR pose considerable technological challenges and if the solutions that are implemented make using the VR tool difficult, excessively complicated or unpleasant, then trainees are unlikely to engage with the simulation (Sutcliffe and Kaur, 2000; Gray, 2002). Questionnaires are typically administered as a means of assessing usability and are often bespoke to the simulation (Sutcliffe and Kaur, 2000).

## Conclusion

In this review we have discussed some of the challenges of validating VR simulations for applications to training. Ultimately, a VR training environment is judged on its ability to elicit positive transfer to the real-world. To achieve this goal, the VR environment needs to be just real enough to develop new skills that can be applied to real tasks. While many realistic behaviors may require the participant to believe they are present in the virtual environment (i.e., place illusion), and that the events are really happening (i.e., plausibility), the perception of presence may not always be the primary consideration when developing effective training simulations^3^. While the concepts of immersion and presence are often used to determine realism, we have suggested expanding this into a typology of fidelity (see Figure 1). Factors such as psychological, affective or ergonomic fidelity are likely to be the more important determinants of effective transfer and are important to evaluate during simulation design. Consequently, researchers are encouraged to address the factors that drive realism in different contexts (e.g., *What is the contribution of each of the constructs in the typology?*) and to explore the extent to which specific markers of fidelity impact upon performance outcomes.

Given the speed of recent technological development it is pertinent to consider what the future of simulated training might look like. One approach that is poised to assume a major role in simulated training is AR and MR, as AR through mobile phone and tablet displays moves into fully immersive headsets, such as the Magic Leap 1 and Microsoft’s HoloLens. AR and MR overlay virtual information on the physical world, which allows real-world training scenarios to be furnished with additional information and guiding cues. AR and MR were not addressed in this framework, but may pose new issues for designing and evaluating training, as fidelity and validity issues may primarily relate to how well the virtual assets are perceived to assimilate with physical ones, and how physical actions interact with virtual assets. Additionally the issue of presence in VR is somewhat avoided, provided that virtual assets are accepted as part of the physical world. Research on AR and MR in training is in its infancy (e.g., see Gavish et al., 2015; Palmarini et al., 2018; Vovk et al., 2018), and further work is needed to explore these issues surrounding testing and validation.

We have provided a number of suggestions for how fidelity and validity can be assessed in VR but also simulated training more generally, and have emphasized the importance of addressing the right markers for the intended training purpose. The potential for VR training is huge, but this field could be hampered by a lack of evidence-based testing and injudicious application. VR technology will continue to develop, driven by the huge gaming market, but a fundamental understanding of the principles underpinning effective design for training purposes and an evidence-based approach to testing will be key to the success of VR training across many domains.

## Author Contributions

DH, JB, PS, MW, and SV contributed to the design, development, and writing of the manuscript.

## Conflict of Interest

The authors declare that the research was conducted in the absence of any commercial or financial relationships that could be construed as a potential conflict of interest.

The reviewer JF declared a past collaboration, with one of the authors, MW, to the handling Editor.

## Footnotes

1 Singley and Anderson (1989) describe the key component for transfer as similarity of the production rule, which specifies the action that must be produced in a certain situation, taking the form of an IF-THEN rule.

2 Similarly, if a test has good construct validity it is likely to show *test-retest reliability*, because it is accurately measuring aspects of the real skill.

3 Presence does appear to correlate with performance in VR, but it is unknown if the same holds true for learning (Stevens and Kincaid, 2015).
